# The Interactions between Insulin and Androgens in Progression to Castrate-Resistant Prostate Cancer

**DOI:** 10.1155/2012/248607

**Published:** 2012-04-03

**Authors:** Jennifer H. Gunter, Amy A. Lubik, Ian McKenzie, Michael Pollak, Colleen C. Nelson

**Affiliations:** ^1^Australian Prostate Cancer Research Centre-Queensland, Queensland University of Technology, Princess Alexandra Hospital, Level 1, Building 1, Ipswich Road, Brisbane, QLD 4102, Australia; ^2^McGill University, Jewish General Hospital, 3755 Côte-Sainte-Catherine Road, Room E-740, Montreal, QC, Canada H3T 1E2

## Abstract

An association between the metabolic syndrome and reduced testosterone levels has been identified, and a specific inverse relationship between insulin and testosterone levels suggests that an important metabolic crosstalk exists between these two hormonal axes; however, the mechanisms by which insulin and androgens may be reciprocally regulated are not well described. Androgen-dependant gene pathways regulate the growth and maintenance of both normal and malignant prostate tissue, and androgen-deprivation therapy (ADT) in patients exploits this dependence when used to treat recurrent and metastatic prostate cancer resulting in tumour regression. A major systemic side effect of ADT includes induction of key features of the metabolic syndrome and the consistent feature of hyperinsulinaemia. Recent studies have specifically identified a correlation between elevated insulin and high-grade PCa and more rapid progression to castrate resistant disease. This paper examines the relationship between insulin and androgens in the context of prostate cancer progression. Prostate cancer patients present a promising cohort for the exploration of insulin stabilising agents as adjunct treatments for hormone deprivation or enhancers of chemosensitivity for treatment of advanced prostate cancer.

## 1. Prostate Cancer and the Metabolic Syndrome

The metabolic syndrome describes a cluster of comorbidities including abdominal obesity, elevated blood glucose, high cholesterol, and hypertension, which increase the risk of developing diabetes and cardiovascular disease [1]. Many of these factors have also been established as risk factors for prostate cancer, strongly suggesting that there is a metabolic component to this malignancy [2–6].

Epidemiological studies have also shown that patients with existing obesity are more likely to be diagnosed with higher-grade cancers and higher Gleason scores, have a higher rate of positive surgical margins at radical prostatectomy, and suffer a higher incidence of prostate cancer recurrence and higher risk of dying of prostate cancer than men with a healthy body mass index (BMI) [7–9]. A number of factors are thought to contribute to these findings including not only a biologic effect by which the endocrine abnormalities of the metabolic syndrome favour aggressive behaviour of prostate cancer but also difficulty in detection as the high rate of benign prostatic hyperplasia (BPH) in obese men increases the likelihood of missing abnormalities with prostate biopsies [10] and misdiagnosis following screening using the prostate-specific antigen (PSA) biomarker; obese men have been reported to have both lower measured PSA, due to increased blood volume and increased PSA concomitant with increased prostate volume and BPH [3], making prostate irregularities complicated to assess.

Dyslipidaemia associated with the metabolic syndrome, including increased triglycerides and LDL and decreased HDL, is also associated with increased prostate cancer risk [11–13], and cholesterol-lowering medications such as statins are currently believed to exert most of their positive effects via systemic reductions in total cholesterol [14]. Both dietary and *de novo* synthesis of free fatty acids have been shown to promote prostate cancer cell survival [15, 16], but studies which characterise fatty acid profiles in prostate disease remain controversial [17, 18].

The metabolic syndrome is associated with altered hormonal profiles for testosterone, insulin, IGFs, and oestrogen, all of which are linked to prostate cancer [19–22]. An association between the metabolic syndrome and reduced testosterone levels exists [9, 23–26] which is not simply related to age [27, 28]. The specific inverse relationship between insulin and testosterone levels observed across the age range from pubertal boys and young men to the elderly (19–90+ years) [26, 28, 29] suggests that an important metabolic crosstalk exists between these two hormonal axes.

## 2. Androgen-Deprivation Therapy and Castrate-Resistant Prostate Cancer

Prostate cancer (PCa) is the most commonly diagnosed lethal cancer in men accounting for approximately one-third of all cancers with a relative lifetime risk of 1 in 7. Its incidence continues to rise with an ageing population, and despite improved survival rates, it remains the second leading cause of cancer deaths in western men [30, 31]. In general, patients with organ-confined PCa are initially treated with radical prostatectomy or radiation-based therapies; however, 25–40% of patients will experience biochemical recurrence defined by a rise in prostate-specific antigen (PSA), an androgen-regulated gene which in these patients acts as a biomarker of recurrent prostate tumour growth and metastatic progression [32]. For decades the most common treatment for metastatic PCa has been androgen-deprivation therapy (ADT) which suppresses testicular testosterone production. Androgen supply is controlled centrally via the hypothalamic-pituitary-testicular axis. Luteinizing hormone-releasing hormone (LHRH) is released from the hypothalamus to activate the anterior pituitary to produce Luteinizing hormone (LH), which stimulates testosterone production from the Leydig cells of the testes. LHRH production is eventually inhibited by the ligand-mediated activation of the androgen receptor [33]. Androgen deprivation is generally achieved using the class of LHRH agonists such as goserelin acetate which disrupts pituitary stimulation and causes dramatic decreases in LH production and subsequent castrate testosterone levels.

Most patients initially respond to ADT; however, after a median 18–36 months patients recur with rising PSA levels despite castrate androgen levels in the serum. This is termed castrate-resistant prostate cancer (CRPC) and leads to significant comorbidities and inevitable mortality [32, 34–36]. The recurrent expression of PSA following the nadir with androgen deprivation therapy implies resumption of androgen receptor activation during progression to castrate resistance. Similarly the fact that up to 30% of patients respond to secondary androgen axis manipulation during castrate resistance implies an ongoing dependence on the androgen receptor pathway in these patients [37]. At least three mechanisms contribute to the reactivation of the androgen receptor in prostate cancer progression. Mutations or splicing events to the ligand binding domain of the androgen receptor (AR) give rise to a promiscuous receptor which permits activation by other molecules including other steroid hormones and antiandrogens (e.g., flutamide) [38]. Amplification of the AR gene has been reported in 30% of tumour samples and is often accompanied by an increase in AR stabilization [35]. A third mechanism followed the observation that, despite low circulating androgen levels with ADT, intraprostatic levels of testosterone in CRPC are high [39]. We and others subsequently demonstrated that, in the face of ADT, prostate tumours upregulate expression of the enzymes necessary to synthesise their own androgens *de novo* [37, 40, 41] resulting in paracrine and autocrine supply of androgens in the prostate tumour microenvironment to sufficient levels to reactivate AR-driven pathways and promote CRPC [40].

Current standard cytotoxic chemotherapies have shown limited benefit for the treatment of CRPC, with modest survival benefits of 2–5 months with docetaxel (Taxotere or cabazitaxel) [42]. The discovery of *de novo* steroidogenesis in prostate tumours as a mechanism driving CRPC has played a significant part in rationalising the newly approved steroidogenic CYP17A1 inhibitor, abiraterone, which is showing great clinical promise for improved control of CRPC [43, 44]. However ~50% of men treated are or ultimately become resistant to abiraterone highlighting the urgent need to understand the factors driving this resistance to develop alternative or adjuvant treatment options [45].

What is striking in patients undergoing ADT is the rapid onset of several key features of the metabolic syndrome in men with no preexisting metabolic dysfunction.

## 3. Androgen Deprivation Causes Metabolic Dysfunction

While ADT is initially an effective treatment for prostate cancer for most patients, the systemic side effects include key features of the metabolic syndrome. Patients typically experience a loss of muscle mass, increased fat mass, and the development of central adiposity, hyperlipidaemia, increased risk of cardiovascular mortality, hyperglycaemia, and the consistent feature of hyperinsulinaemia [46–48]. These in turn are associated with poor outcomes including more rapid progression to advanced disease and increased cancer mortality [6, 49, 50]. Moreover, recent studies have specifically identified a correlation between elevated insulin/C-peptide levels (normalized surrogate insulin levels) with high-grade PCa and worse patient prognosis [6, 9, 47, 51–54]. Pre-diagnostic body mass index (BMI) and C-peptide correlate with the risk of prostate cancer-specific mortality, and obese men (BMI > 30) were more likely to have extraprostatic or metastatic prostate cancer, or a higher Gleason grade of cancer at the time of diagnosis than men with BMI < 30. Of greater significance, patients with higher C-peptide had an increased prostate cancer-specific mortality compared to those with low C-peptide levels suggesting that at least part of the effect of increased BMI on mortality was related to coincident hyperinsulinaemia [6]. Similarly, a nested case-control trial within the Prostate Cancer Prevention Trial found that while increasing C-peptide level was weakly associated with cancer risk, there was a strong association with the development of high-grade prostate cancer of Gleason grade 7 or greater. In contrast to the Ma study, this association was found to be independent of BMI [54]. J. Hammarsten and B.Högstedt prospectively assessed baseline insulin levels at time of prostate cancer diagnosis and compared them between men who died from prostate cancer during 5 years of followup and men who survived. Statistically significant risk factors identified for lethal prostate cancer included both type 2 diabetes and hyperinsulinaemia. But only hyperinsulinaemia remained significant after adjusting for stage and grade of prostate cancer, factors known to independently affect prognosis, strongly suggesting that hyperinsulinaemia is the key promoter of prostate cancer progression associated with metabolic dysfunction [53]. Thus, while ADT initially is effective treatment for prostate cancer, the metabolic complications of ADT may not only cause multisystem morbidity (obesity, increased risk of stroke, and so forth) but also lead to an altered hormonal environment that favours the development of castrate-resistant behaviour.

## 4. Chicken or the Egg: The Interrelationship between Insulin and Testosterone

While we have just discussed the increased insulin resistance and hyperinsulinaemia which result from pharmacological inhibition of testicular testosterone production, it is also true that pre-existing hyperinsulinaemia, such as that seen in type II diabetes, is associated with reduced testosterone levels. The inverse relationship between testosterone and insulin in males without cancer has been well documented; yet the mechanisms linking these two hormonal pathways remain poorly understood [24, 55, 56].

Reduced levels of free and total testosterone have been associated with type 2 diabetes (T2DM), central adiposity, dyslipidaemia, and hyperinsulinaemia in various studies [24–26, 57–61], usually in combination with reduced LHRH and LH levels however, the observation of low testosterone levels remains relatively rare in men with type 1 diabetes suggesting that hyperglycaemia is not a direct cause. In addition, low androgen levels are observed in lean men with T2DM suggesting that raised BMI is not necessary for the persistence of hypogonadism [62]. In population studies, following adjustment for age and adiposity, insulin was found to be significantly and inversely correlated to free and total testosterone levels [24]. Further, a study of middle-aged men followed over 11 years found that low testosterone levels were predictive for development of the metabolic syndrome and heralded as a predictive biomarker of metabolic and diabetic pathogenesis [58]. In contrast, high testosterone levels are linked to insulin sensitivity, and pharmacological improvements to insulin sensitivity increase testosterone levels [63, 64].

Several mechanisms have been proposed which may contribute to reduced testosterone/insulin resistance in these cohorts. Testosterone can be converted to oestradiol through the irreversible action of aromatase in adipose tissue. Excess adipose tissue in obesity may increase the rate of conversion of testosterone to oestradiol, a more potent inhibitor of LHRH secretion from the hypothalamus. Studies investigating the levels of oestradiol in these patients, however, show mixed results. Decreased testosterone is coupled with increased oestradiol in some studies [65, 66] while others have shown reduced oestradiol concentrations in hypogonadal men [67, 68], consistent with reduced testosterone substrate. Differences in the sensitivity of detection methods and duration of condition may account for discrepancies in these reports. Hypogonadism as a result of age or in lean men with type 2 diabetes is likewise associated with reduced levels of oestradiol suggesting that decreased testosterone levels in these men is not likely to be due to oestradiol-mediated suppression of LHRH secretion [62, 68].

LHRH and LH secretion is suppressed in animal models by increased circulating cytokines which are elevated in obesity [69, 70]. Related changes to the adipokine secretion profile of men receiving ADT may also contribute to reductions in insulin sensitivity [47, 71]. Leptin and adiponectin, circulating factors secreted by adipose tissue with known modulatory functions on insulin sensitivity, are both elevated during ADT [71, 72]. Leptin plays an important role in appetite and energy balance, immune modulation, and bone homeostasis [73] and is secreted from fat tissue in proportion to adiposity. Circulating levels of leptin are increased during ADT, in line with increased deposition of fat in these men; however, leptin has been shown to increase even in the absence of discernable weight gain following 28 days of ADT, although relative fat/lean mass was not measured in this study [71]. The profile of adiponectin secretion is generally opposite to leptin with reduced expression and secretion with increasing adiposity [74]. However, adiponectin is normally suppressed by testosterone [75–77], and the resulting increase in adiponectin following ADT is attributed to the loss of suppression by androgen. The insulin-sensitising effects of adiponectin which may be present following ADT, however, fail to overcome the effects of androgen withdrawal on the development of hyperinsulinaemia [71, 72].

A recently published paper by Rubinow et al. manipulated testosterone levels in young–middle-aged healthy men, excluding patients with confounding underlying conditions such as prostate cancer, diabetes, and hypogonadism [71]. Groups were randomised to receive ADT alone, ADT with testosterone replacement, and ADT with testosterone replacement and an aromatase inhibitor. Decreased insulin sensitivity was observed in the men receiving ADT only, in line with >90% reduction in circulating testosterone levels which was observed in the absence of changes to body weight and fasting glucose concentrations. Insulin sensitivity was not affected by reduced oestradiol levels in participants receiving the aromatase inhibitor suggesting that testosterone is the major regulator of insulin sensitivity in healthy males. Similar observations have been made of men receiving ADT for prostate cancer. The rapid withdrawal of androgens with ADT causes hyperinsulinaemia and loss of insulin sensitivity in these patients, reflected by increased homeostatic model assessment (HOMA) score, within 2 weeks [78] suggesting that this is a direct effect of ADT and not subsequent to changes in fat mass. On the contrary, studies have shown that insulin sensitivity following ADT is independent of fat mass and age [48, 79].

These observations provide strong evidence for an important functional role for hyperinsulinaemia in PCa progression and CRPC following androgen deprivation. Major findings from recent studies [54, 80] of men receiving ADT demonstrated a strong trend between an elevated C-peptide level and more rapid progression to CRPC. Although there is mounting epidemiological evidence linking hyperinsulinaemia and CRPC, the mechanisms of insulin action directly on PCa cells in the context of ADT has, until recently, not been the subject of biological scrutiny.

## 5. Potential Mechanisms of Insulin-Androgen Crosstalk in the Prostate

**Figure 1:**
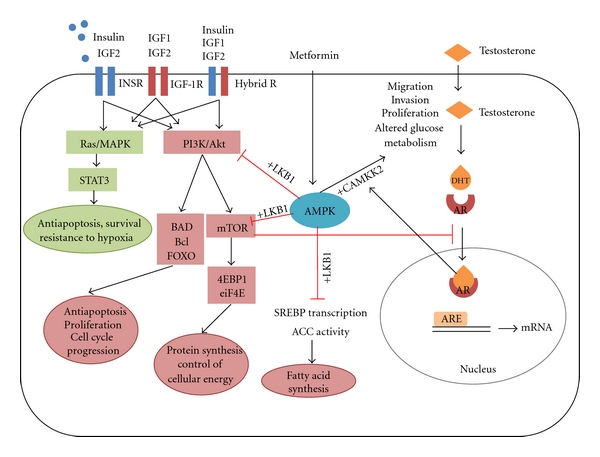
Insulin receptor (INSR) belongs to a family of receptor tyrosine kinases that includes the receptor for insulin-like growth factor 1 (IGF-1R). Many tumour types have upregulated expression of IGF-1R, INSR, and potentially hybrid INSR/IGF-1Rs which facilitate increased activation of mitogenic, prosurvival and protein synthesis pathways following activation by of ligands insulin, IGF-1 or IGF-2. IGF-1 can bind the INSR with 2.5% the efficiency of insulin. Insulin, at physiological levels, will not activate the IGF-1R. Insulin and IGF-1 can activate signalling with varying potency through the hybrid INSR/IGF-1R. IGF2 is able to signal through each of the INSR, IGF-1R, and hybrid receptor (Hybrid R). Ligand binding to the INSR and IGF-1R activate numerous downstream pathways including phosphatidylinositol 3 kinase (PI3K)/Akt and Ras/MAPK pathways with many well-characterised downstream effects including increased glucose metabolism, inhibition of apoptosis (e.g., via FOXO and BAD-mediated pathways), and stimulation of cell proliferation (e.g., via mammalian target of rapamycin; mTOR). Reciprocal feedback regulation occurs in prostate cancer cells between the AR signalling and signalling through the PI3K-AKT-mTOR pathway. Unfettered activity through this pathway, associated with the common PTEN mutation, inhibits AR signalling and suggests a possible pathway to androgen-independent growth of prostate tumours. AMP-activated protein kinase (AMPK) is a potentially bifunctional modulator in prostate cancer cells. Activation of AMPK modulates insulin signalling by negatively regulating mTOR control of protein synthesis and halting cell proliferation. AMPK activation also promotes fatty acid oxidation and downregulates expression of SREBP and activity of acetyl-CoA carboxylase (ACC) resulting in decreased fatty acid synthesis. However, AMPK has complex regulation in prostate cancer cells and may potentiate *increased *proliferation and migration when activated downstream of the AR under the control of AR responsive regulator calcium/calmodulin-dependent protein kinase kinase 2 (CAMKK2). AR directly regulates CAMKK2 and upregulates AMPK which mediates AR-induced migration and invasion in a CAMKK2-dependent manner. In studies where metformin activation of AMPK results in cessation of cancer cell growth, signalling is thought to be through LKB-1 tumour suppressor; therefore, competition for AMPK signalling via LKB1 stimulation versus AR-mediated CAMKK2 activation could result in altered functional outcomes. DHT, dihydrogen testosterone; AR, androgen receptor; ARE, androgen response element; MAPK, mitogen-activated protein kinase.

Traditionally insulin has been primarily considered a hormone essential for metabolic regulation; however, insulin can also activate lipogenesis, steroidogenesis, protein synthesis and antiapoptotic survival pathways in many cell types [81, 82]. Insulin positively and negatively regulates approximately 150 genes; however, transcriptional factors act differently in different target tissues; thus, insulin affects transcription by modulation of the level, localization, and activity of transcription factors differently in specific microenvironments [82]. Insulin signals through its cognate receptor of which there are two isoforms, INSR-A and INSR-B [81], that belong to a family of receptor tyrosine kinases that includes the receptor for insulin-like growth factor 1 (IGF-1R). Many tumour types have upregulated expression of IGF-1R, INSR, and potentially hybrid INSR/IGF-1Rs which facilitate increased activation of mitogenic, prosurvival and protein synthesis pathways with the increased levels of ligands insulin, IGF-1, or IGF-2 [83–85]. IC_50_ values, calculated for each ligand and each receptor, reveal that IGF-1 can bind the INSR-A with ~2.5% the efficiency of insulin and with even weaker affinity to INSR-B. Likewise, insulin, at physiological levels, will not activate the IGF-1R [81]. In contrast, insulin and IGF-1 can activate signalling with varying potency through the hybrid INSR/IGF-1R. IGF-2 is able to signal through each of the INSR-A (weakly binds INSR-B) and IGF-1R as well as hybrid INSR/IGF-1R. Ligand binding to the INSR and IGF-1R activate numerous downstream pathways including phosphatidylinositol 3-kinase (PI3K)/Akt and Ras/MAPK pathways with many well-characterised downstream effects including increased glucose metabolism, inhibition of apoptosis (e.g., via FOXO and BAD-mediated pathways) and stimulation of cell proliferation (e.g., via mTOR) (Figure 1) [81].

While the role of IGF-1 in cancer has been recognised for over 20 years, the presence of the INSR directly on prostate tumour tissue has only recently been reported and show that increased INSR expression correlates with increasing Gleason grade and CRPC [51] providing further evidence that insulin and insulin receptor signalling may have a critical role driving progression of advanced prostate cancer. As prostate epithelial cells are not subject to development of insulin resistance, defined by impaired glucose uptake and metabolism, as occurs in insulin-sensitive metabolic tissue, hyperinsulinaemia would be expected to increase insulin signalling in PCa cells [81] in parallel to the elevated levels of ligand.

The molecular bases for the inverse clinical observations between levels of insulin and testosterone are less well described. The androgen receptor is a member of the steroid hormone receptor family and classically controls transcription of androgen-regulated genes in a ligand-dependent manner; however, androgens can also elicit rapid signalling responses independent of the AR [86, 87]. Androgen receptor signalling plays a role in metabolic function. Direct AR-mediated effects of testosterone on fat metabolism are evidenced by AR knockout mice which have increased adiposity, accompanied by elevated leptin and adiponectin profiles and altered lipid metabolism [88], and men with genetic androgen resistance due to defective AR expression have increased central adiposity [89]. AR signalling in muscle tissue may also affect systemic insulin sensitivity. AR activation favours development of pluripotent stem cells down the myogenic lineage via AR-dependent activity through noncanonical Wnt signalling, favouring the formation of muscle while suppressing the formation of new fat tissue (adipogenesis) [90]. AR activation in muscle tissue also increases oxidative metabolism and insulin sensitivity via upregulation of the transcription factor PPAR*γ* coactivator 1*α* (PGC1*α*) which stimulates mitochondrial biogenesis and increases the oxidative potential of skeletal muscle; decreased testosterone levels are associated with decreased PGC1*α* levels and increased insulin resistance. The activation of PGC1*α* presents a potential mechanism for cancer cell survival that may confer increased resistance to oxidative stress and cellular senescence by increasing oxidative phosphorylation in the tumour cell [91, 92].

Recent reports have identified previously unappreciated crosstalk in prostate cancer cells between the AR pathway and PI3K signalling pathway, the major signalling pathway activated downstream of the INSR [93–96]. The PI3K pathway has been implicated in a number of malignancies [97] including prostate cancer. Approximately 40% of primary and 70% of metastatic prostate cancers have mutations within the PI3K signalling pathway, mostly associated with a loss of the negative regulator, PTEN [98–100]. Reciprocal feedback regulation between androgen receptor signalling and unfettered signalling through the PI3K-AKT-mTOR pathway in prostate cancer [93, 95] have been shown to inhibit AR signalling and suggest a possible pathway to androgen-independent growth of prostate tumours [93], and conversely, inhibition at each of these signalling nodes was associated with enhanced AR signalling and increased transcription of AR-responsive genes (Figure 1).

Hyperinsulinaemia and increased insulin signalling in prostate tumour cells as a result of androgen deprivation are likely to activate survival pathways downstream of the insulin receptor which have the potential to contribute to progression to castrate resistance; thus, these candidate molecules downstream of insulin receptor signalling may have therapeutic utility in advanced prostate cancer.

## 6. Diabetes, Prostate Cancer, and Metformin

Diabetes has been shown to be associated with increased risk of several cancers including colon, pancreatic, and breast cancer [101]. In contrast studies have found that diabetics have lower PCa risk than nondiabetics [102, 103], perhaps related to decreased levels of androgens [103–105]. Metformin, which works in part by activating AMP-activated protein kinase (AMPK), is used clinically in obese and diabetic patients to normalise circulating insulin levels primarily via reduced hepatic glucose output, and promising data emerging from clinical studies suggests that metformin may improve patient outcomes in prostate and other cancers [106–110]. A retrospective cohort study compared diabetic patients on metformin against matched diabetic patients not receiving metformin and showed a significantly decreased risk of cancer diagnosis of the metformin group with an adjusted hazard ratio of 0.63 (95% CI 0.53–0.75) and decreased cancer-related mortality, as well as a prolonged median time to cancer diagnosis [111]. These results were supported by an observational study of 11,876 diabetic patients demonstrating a 33% decreased risk of developing cancer with metformin treatment compared to other treatments [112]. In contrast, metformin gave no benefit to prostate cancer risk compared to controls in a recent report; on the contrary, metformin treatment was reported to increase the risk of prostate cancer diagnosis back up to normal levels [113] possibly due to the normalisation of androgen levels associated with stabilisation of insulin levels. 

Based on these results, targeting AMP-activated protein kinase (AMPK) has been proposed as a therapeutic strategy in cancer [114, 115]. Reports indicate that pharmacological activation of AMPK in cancer cells by either metformin or AICAR results in halting cell proliferation by negatively regulating mammalian target of rapamycin (mTOR) control of protein synthesis (Figure 1) [101, 116–118]. *In vitro* studies of metformin also demonstrate an antitumoral effect in prostate cancer cells by blocking cell cycle progression and decreasing cyclin D1 protein levels resulting in reduced LNCaP xenograft tumour growth [107]. AMPK activation also promotes fatty acid oxidation reducing availability of fatty acids for biosynthetic pathways and downregulating expression of SREBP and phosphorylation/deactivation of acetyl-CoA carboxylase (ACC) resulting in decreased fatty acid synthesis (Figure 1) [119].

Activation of AMPK modulates insulin signalling downstream of the insulin receptor [120], most notably via differentially phosphorylating the tuberous sclerosis complex TSC1-TSC2 to inactivate mTOR [118, 121]. The ability of AMPK to potentiate insulin action on cancer cell growth and survival has not been greatly explored in models of prostate cancer. Indeed the effects of metformin have not yet been demonstrated to be direct effects on cancer cells or an indirect consequence of systemic insulin normalisation [115]. Nevertheless, the intersection between mTOR, insulin signaling, and AMPK provides an intriguing, tantalising link between cellular energy and cancer pathways.

Recent data suggests that AMPK activation may have more complex regulation in prostate cancer cells and may potentiate increased prostate cancer cell proliferation and migration when activated downstream of the androgen receptor (AR) [122, 123]. Using elegant integrated bioinformatic analyses of transcriptional profiling with AR ChIP analysis, Massie et al. recently identified AR-mediated upregulation of several metabolic pathways including increased aerobic glycolysis under the control of a master regulator calcium/calmodulin-dependent protein kinase kinase 2 (CAMKK2) [123]. AR directly regulates CAMKK2 by binding to its promoter and is highly expressed in normal prostate with elevated expression in both AR-sensitive and CRPC models of prostate cancer [122, 123]. Intriguingly, AR activation was also shown to upregulate the primary CAMKK2 substrate AMPK [122–124] which mediated AR-induced migration and invasion in a CAMKK2-dependent manner (Figure 1). Massie et al. found that CAMKK2 activation of AMPK promoted glycolysis but negatively regulated biosynthesis though mTOR pathways consistent with previous reports of AMPK inhibitory effect on components of the mTOR pathway [117, 118]. Similarly, in an earlier report, activation of AMPK was sufficient to increase cell migration via potentiation of Rac1 activity, a regulator of cell migration [122, 125]. Alternative cofactors yet to be identified may regulate this effect of AMPK, specifically downstream of AR signalling.

In studies where metformin activation of AMPK results in cessation of cancer cell growth, signalling is thought be through LKB-1 tumour suppressor [126]; therefore, the difference in these functional outcomes of AMPK activation could be the company it keeps. Competition for AMPK signalling via LKB1 stimulation versus AR-mediated CAMKK2 activation could result in decreased mTOR signalling and decreased glucose (fuel) for anabolic pathways. Taken together, it seems that AMPK at the cellular level is a potentially bi-functional modulator. This latter role for AMPK is in contrast to earlier reports which show that metformin reduced cancer cell proliferation [107, 117, 127, 128] via inhibition of anabolic pathways such as lipogenesis [119, 129] starving the major bioenergetic pathway in prostate cancer cells [130]. Nevertheless, we should interpret with caution the effect of metformin from men with pre-existing obesity or type 2 diabetes where, in contrast to other cancers, diabetes appears to protect from cancer, while metformin may increase the risk of diagnosis or more likely mitigate the PCa risk-reducing benefit of diabetes in these patients. Several clinical trials are currently addressing the benefit of metformin treatment in men on ADT without pre-existing diabetes. The outcomes of these trials will be of significant clinical interest.

## 7. Targeting the Insulin Axis in Advanced Prostate Cancer

Reactivation of the AR following ADT, heralded by rising serum PSA, is a hallmark of CRPC progression. We and others have demonstrated in recent years that intratumoral androgen synthesis is a major contributor to reactivation of AR in CRPC [39, 40, 131, 132]. Moreover, we have noted that insulin promotes steroidogenesis in other cell types [133–135]. From these merging hypotheses, we have recently reported that insulin, in the absence of androgen, may drive PCa progression in part through upregulation of SREBP and its downstream enzymes responsible for lipid and steroid synthesis in cell models of prostate cancer, resulting in dramatically increased intratumoral steroid production which is sufficient to reactivate the AR to stimulate PSA expression and secretion [136]. Thus, insulin can act directly on PCa cells to activate pathways contributing to CRPC progression.

In addition to activation of *de novo* steroid synthesis, insulin is capable of driving numerous transcriptional programs predominantly downstream of PI3K/AKT signalling. Pathways activated in response to cell stress (e.g., glucose starvation, hypoxia) are associated with increased cancer cell survival, via both pro-proliferative and antiapoptotic mechanisms and underpin treatment resistance [137–140]. 

Many of these pathways are modulated by highly conserved signalling molecules including insulin, target of rapamycin (mTOR) and AMP-activated protein kinase (AMPK), each of which maintain cellular homeostasis by sensing/signalling nutrient, energy, and oxygen availability. These molecules integrate cellular energy and metabolism with stress response pathways leading to cancer cell survival. More recently, the insulin-sensitising class of drugs, thiazolidinediones, has also been associated with improved survival of diabetic prostate cancer patients [63] revealing several potential nodes of therapeutic intervention which warrant further research.

## 8. Conclusions

Prostate cancer is the most common cancer in men [30], increases sharply after age 50, and will continue to rise with our ageing population. At the same time, we face the growing epidemic of obesity and associated metabolic syndrome while the risk of aggressive prostate cancer is increased 3-fold with obesity. Therapy for local control will fail in 25–40% of prostate cancer patients, and these men will subsequently be treated with ADT [32]. While initially improving cancer control, ADT induces hyperinsulinaemia [32, 34–36]. We have shown that insulin acts directly on prostate tumour cells to increase intratumoural androgen production. However, it is likely that additional highly relevant cancer pathways are activated by high insulin levels that promote metastases, tumour growth, and treatment resistance and that the crosstalk between these pathways and AR signalling may be highly relevant to the progression to castrate-resistant disease, possibly independently of AR signalling.

Standard chemotherapeutic agents have limited benefit in CRPC and while next-generation anti-androgen therapies are improving, they still result in resistance, highlighting the urgent need to understand mechanisms underlying treatment resistance and find rationally informed treatment options. Currently, ADT-induced hyperinsulinaemia is not addressed in prostate cancer patients, despite a significantly increased risk of cardiovascular and cancer-related mortality in these patients [141]; however, a review of the literature suggests that management of the hyperinsulinaemia induced by ADT may be a useful adjunct to current standard prostate cancer treatments.
